# Meta-analysis of 8q24 for seven cancers reveals a locus between *NOV *and *ENPP2 *associated with cancer development

**DOI:** 10.1186/1471-2350-12-156

**Published:** 2011-12-05

**Authors:** Abra G Brisbin, Yan W Asmann, Honglin Song, Ya-Yu Tsai, Jeremiah A Aakre, Ping Yang, Robert B Jenkins, Paul Pharoah, Fredrick Schumacher, David V Conti, David J Duggan, Mark Jenkins, John Hopper, Steven Gallinger, Polly Newcomb, Graham Casey, Thomas A Sellers, Brooke L Fridley

**Affiliations:** 1 Department of Health Sciences Research, Mayo Clinic, Rochester, USA; 2 Department of Oncology, Strangeways Research Laboratory, University of Cambridge, Cambridge, UK; 3 Department of Cancer Genetics and Epidemiology, Risk Assessment, Detection, and Intervention Program, Moffitt Cancer Center, Tampa, USA; 4 Division of Experimental Pathology, Mayo Clinic, Rochester, USA; 5 Department of Preventive Medicine, University of Southern California, Los Angeles, USA; 6 Translational Genomics Research Institute, Phoenix, USA; 7 School of Population Health, University of Melbourne, Victoria, Australia; 8 Samuel Lunenfeld Research Institute, Mount Sinai Hospital, Toronto, Canada; 9 Fred Hutchinson Cancer Research Center, Seattle, USA

## Abstract

**Background:**

Human chromosomal region 8q24 contains several genes which could be functionally related to cancer, including the proto-oncogene c-MYC. However, the abundance of associations around 128 Mb on chromosome 8 could mask the appearance of a weaker, but important, association elsewhere on 8q24.

**Methods:**

In this study, we completed a meta-analysis of results from nine genome-wide association studies for seven types of solid-tumor cancers (breast, prostate, pancreatic, lung, ovarian, colon, and glioma) to identify additional associations that were not apparent in any individual study.

**Results:**

Fifteen SNPs in the 8q24 region had meta-analysis p-values < 1E-04. In particular, the region consisting of 120,576,000-120,627,000 bp contained 7 SNPs with p-values < 1.0E-4, including rs6993464 (p = 1.25E-07). This association lies in the region between two genes, *NOV *and *ENPP2*, which have been shown to play a role in tumor development and motility. An additional region consisting of 5 markers from 128,478,000 bp - 128,524,000 (around gene *POU5F1B*) had p-values < 1E-04, including rs6983267, which had the smallest p-value (p = 6.34E-08). This result replicates previous reports of association between rs6983267 and prostate and colon cancer.

**Conclusions:**

Further research in this area is warranted as these results demonstrate that the chromosomal region 8q24 may contain a locus that influences general cancer susceptibility between 120,576 and 120,630 kb.

## Background

Human chromosomal region 8q24 has been associated with many types of solid-tumor cancer, including cancers of the breast [1-5], prostate [6-10], bladder [11], colon [12-17], lung [18,19], ovaries [20], pancreas [21], and brain [22,23] (Additional file 1). The majority of these associations lie at approximately 128 Mb on chromosome 8, with one prominently associated SNP, rs6983267, shown to interact with the proto-oncogene c-MYC (128.82 Mb) [1,24]. Several studies suggest the possibility that some loci in 8q24 influence more than one type of cancer per locus: breast and pancreatic cancer [2]; prostate and colorectal cancer [25]; prostate, colorectal, and ovarian cancer [20]. These studies suggest that this region may contain loci that affect general cancer susceptibility, which interact with other loci (in or outside of 8q24) and/or environmental factors to determine cancer type.

Therefore, to fully understand the role of 8q24 in cancer development, it is important to determine any additional associations that lie within this region. For example, the region 8q24 contains several other genes which could be functionally related to cancer development, including *NOV*, which encodes a regulatory protein from the CCN family that has been associated with cancer development [26]. Therefore, we used data from nine genome-wide association studies (GWAS) for seven cancers to conduct a meta-analysis for general cancer risk loci in the entire region of 8q24.

## Methods

### Nine Genome-Wide Association Studies for Cancer

The meta-analysis consisted of solid-tumor cancer GWAS with data available via dbGaP or available to us through collaborations, including studies of lung [27], prostate [28], breast [29,30], and pancreatic [31] cancers from the Cancer Genetic Markers of Susceptibility (CGEMS) project, accessed from dbGaP; an additional lung cancer study [32]; the Cancer Family Registry (CFR) colon cancer study [33], a study of glioma [34], and two ovarian cancer studies from the United Kingdom [35] and the United States [36]. Table 1 presents a summary of the nine studies. The reader is referred to the primary papers for more extensive study details regarding recruitment, matching of controls and analysis methods.

**Table 1:** Summary of the nine GWAS included in the meta-analysis for 8q24.

Cancer	Ref	Study	Study size	Cases	Controls	Cancer subtypes	Study Notes
Ovarian	[35,38]	UK	4170	1817	2353		Majority of controls from UK 1958 birth cohort
Ovarian	[36]	US	3715	1815	1900	epithelial ovarian cancer	Four incident case-control studies: Mayo Clinic Ovarian Cancer Study, Duke University's North Carolina Ovarian Cancer Study, University of Toronto Familial Ovarian Tumor Study, H. Lee Moffitt Cancer Center and Research Institute's Tampa Bay Ovarian Cancer Study
Glioma	[34]	Mayo Clinic	350	176	174	high-grade glioblastoma, anaplastic astrocytoma	
Lung	[32]	Mayo Clinic	754	377	377	adenocarcinoma, other	Cases and controls were never smokers (i.e., had smoked fewer than 100 cigarettes in their lifetime)
Lung	[27]	CGEMS/PLCO	1629	~800	~800	adenocarcinoma, squamous cell carcinoma, small-cell lung cancer, other	Cases and controls consisted of both smokers & non-smokers
Prostate	[28]	CGEMS/PLCO	2252	1151	1101	aggressive, non-aggressive	
Breast	[29,30]	CGEMS/Nurses' Health Study	2287	1145	1142		Postmenopausal women with matching based on use of hormone replacement therapy
Pancreatic	[31]	CGEMS/PanScan	7174	3532	3642	primary adenocarcinoma of the exocrine pancreas	Consisted of 12 cohort and 8 case-control studies
Colon	[33]	Colon Family Registry (CFR)	2190	~1000	~1000	invasive, microsatellite stable or low microsatellite instability colorectal cancer	Cases had no germline mismatch repair mutations

The CGEMS lung cancer GWAS [27] consisted of 1629 subjects from the Prostate, Lung, Colorectal, and Ovarian (PLCO) cancer study who provided consent for general cancer research. The study included adenocarcinoma, squamous cell carcinoma, small-cell lung cancer, and other histological types. The second lung cancer GWAS included 377 lung cancer cases and 377 matched controls, all of whom were never smokers (i.e., had smoked fewer than 100 cigarettes in their lifetime) [32]. Eighty-one percent of the cases had non-small-cell lung cancer (NSCLC); 68% had adenocarcinoma, a type of NSCLC. The CGEMS prostate cancer GWAS [28] included 1151 cases and 1101 male controls from the screening arm of the PLCO study. The cases included individuals with aggressive and nonaggressive cancer. The CGEMS breast cancer GWAS [29,30] consisted of 1145 cases and 1142 matched controls, all of whom were postmenopausal women of European ancestry from the Nurses' Health Study. The fourth CGEMS GWAS included was the pancreatic cancer GWAS [31], which included 7174 subjects drawn from cohort and case-control studies. All cases had primary adenocarcinoma of the exocrine pancreas.

The CFR colon cancer GWAS [33] consisted of 2190 individuals. The cases had invasive colorectal cancer and no identified germline mutations in mismatch repair proteins. The cases were self-identified as non-Hispanic white and had microsatellite stable or low microsatellite instability colorectal cancer (CRC) and/or MMR protein immunohistochemistry positive determined using standard methods [37]. The glioma GWAS [34] included 176 cases and 174 controls from the Mayo Clinic. The cases were adults with high-grade glioblastoma or anaplastic astrocytoma. Finally, the UK ovarian cancer GWAS [35,38] involved 1817 cases and 2353 controls, while the US ovarian GWAS [36] included 1,815 cases and 1,900 controls from four epithelial ovarian cancer (EOC) case-control studies.

### Meta-analysis

The meta-analysis included 6686 SNPs across the 8q24 region, from rs6469653 (117.7 Mb) to rs7822726 (146.2 Mb), which were genotyped and/or imputed in at least five studies. P-values from an additive genetic model for the nine cancer GWAS were combined using Fisher's method [39] and Stouffer's (Lipták) method [40], which each use different transformations for combining *m *independent p-values (*p_i_*, *i *= 1, ..., *m*) into a test statistic. In particular, the test statistic for Fisher's method is $F=-2\overset{m}{\underset{i=1}{\sum }}\log\left(p_{i}\right)$, while the test statistic for Stouffer's method is $S=\overset{m}{\underset{i=1}{\sum }}Z_{i}/\sqrt{m}$, with *Z_i _*= Φ^-1^(*p_i_*), where Φ^-1^(·) is the inverse standard normal cumulative distribution function. Loughin [41] recommended that Fisher's method be used to emphasize small p-values, while Stouffer's method is preferable for putting equal weight on p-values at both extremes. Therefore, for the meta-analysis both Fisher's and Stouffer's methods were used to assess robustness of the findings.

Many meta-analyses weight results by the sample size of the studies included [42]. This practice is useful for meta-analyses based on averaging effect sizes, as larger studies are expected to have more accurate estimates of effect sizes [43]. However, meta-analyses based on p-values, such as this one, already incorporate sample size, as smaller studies contain less evidence and cannot have extremely small p-values. Simulations demonstrate that further weighting the analysis by sample size can bias the results toward the largest study and decrease power to detect effects shared only among the smaller studies (Additional file 2). For this reason, equal weight was given to each cancer type in the meta-analysis. Each of the two lung cancer studies and each of the two ovarian studies received a weight of one-half. The Fisher's meta-analysis was weighted according to the method of Hou [44].

In addition to the meta-analysis based on combination of p-values, analysis was also completed based on the number of individual studies in which the SNP had an individual association p-value less than 0.10, with the p-value based on a binomial distribution conditioned on the number of studies in which each SNP was genotyped. Linkage disequilibrium (LD) plots and statistics for Europeans (CEU) were derived from HapMap release 27 (http://hapmap.ncbi.nlm.nih.gov/downloads/ld_data/latest/; [45]). LocusZoom (http://csg.sph.umich.edu/locuszoom/; [46]) was used to plot association results.

## Results

**Figure 1:**
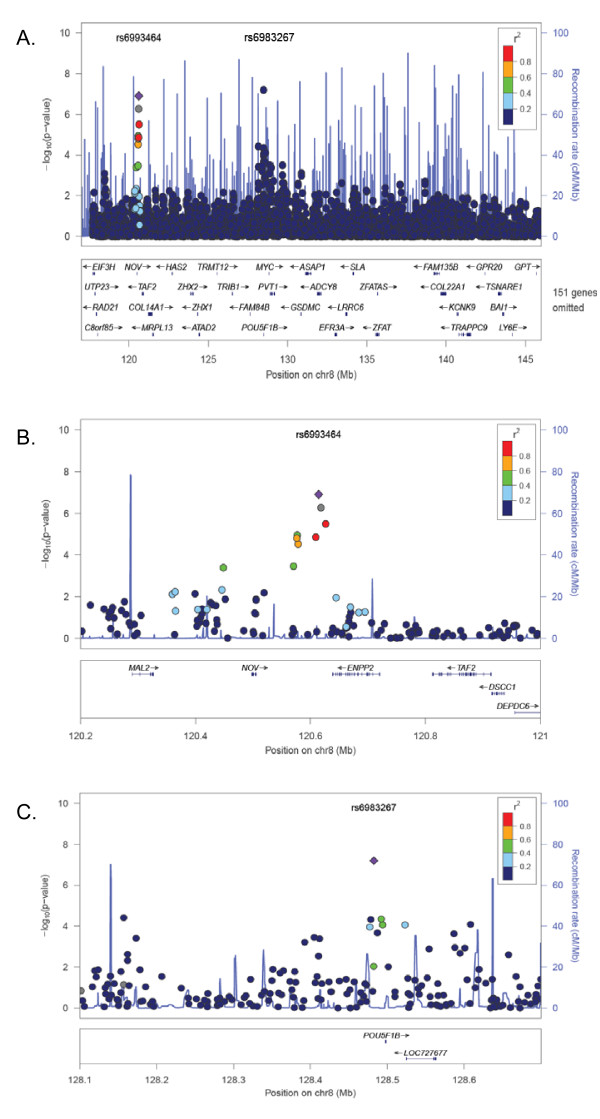
**Individual GWAS top results and meta-analysis results for 8q24 region**. (A) -log_10_(p-values) from meta-analysis using Fisher's method of the 8q24 region; (B) -log_10_(p-values) from meta-analysis for the region around *NOV *and *ENPP2*. Purple diamond = rs6993464; gray = no LD data available in HapMap Phase II. Other colors indicate level of LD with rs6993464. (C) -log_10_(p-values) from meta-analysis using Fisher's method for the region around rs6983267. Purple diamond = rs6983267; colors indicate level of LD with rs6983267.

Fifteen SNPs had meta-analysis p-values below 1E-04, as shown in Table 2. The region chr8: 120,576,000-120,627,000 bp contains 7 SNPs with meta-analysis p-values < 1.0E-5 (Figures 1A and 1B), including rs6993464 (Fisher's p = 1.25E-07), which had the second-most significant p-value of the 6686 SNPs examined within 8q24. This p-value is significant after Bonferroni correction (p = 0.0025). In addition, two of the 7 SNPs had Stouffer's p-values < 1E-04, including rs6993464 (Stouffer's p = 6.92E-07). The agreement between the Fisher and Stouffer results demonstrates robustness of the result to the choice of transformation used in combining the p-values.

**Table 2:** Top meta-analysis results.

		Individual Study P-value									Meta-analysis P-value		
SNP	bp	Breast^1^	Prostate^1^	Pancreatic^1^	Lung^1^	Lung^2^	Ovarian^3^	Ovarian^4^	Glioma^5^	Colon^6^	Fisher	Stouffer	Binomial
rs1695718	118458790	0.14	0.40	0.82	0.19	-	0.95	0.03	**5.45E-05**	0.23	7.94E-04	8.92E-03	0.19
rs7846200	120576453	1.08E-03	0.18	7.52E-04	6.20E-03	-	0.87	0.61	0.40	0.39	**1.51E-05**	1.01E-04	0.04
rs7000665	120576919	1.73E-03	0.22	1.12E-04	8.44E-03	-	0.90	0.54	0.62	0.45	**1.11E-05**	2.39E-04	0.04
rs10505364	120578285	1.53E-03	0.27	2.97E-04	3.16E-03	0.75	0.82	0.46	0.37	0.39	**2.94E-05**	3.72E-04	0.05
rs9643136	120609324	5.45E-04	0.34	1.15E-04	5.30E-03	0.50	0.99	0.58	0.70	0.36	**1.36E-05**	1.78E-03	0.05
rs6993464	120614129	2.17E-03	0.17	**6.39E-05**	6.45E-04	-	0.81	0.22	0.21	0.24	**1.25E-07**	**6.92E-07**	0.04
rs11782176	120617858	2.16E-03	0.20	**5.55E-05**	5.61E-04	-	0.76	-	0.24	0.25	**5.24E-07**	**6.86E-06**	0.03
rs6991756	120626148	4.33E-03	0.24	**5.01E-05**	1.15E-03	1.00	0.69	0.24	0.24	0.23	**3.17E-06**	1.34E-04	0.05
rs3870371	122766313	**4.23E-05**	0.57	0.62	0.38	0.82	0.23	0.16	0.69	0.72	9.12E-03	0.12	0.61
rs920455	122769378	**3.30E-05**	0.65	0.64	0.41	0.68	0.16	0.18	0.85	0.76	9.26E-03	0.17	0.61
rs6470494	128157086	9.43E-04	0.36	0.16	0.96	0.57	2.88E-03	0.93	0.57	5.85E-04	**3.73E-05**	1.60E-03	0.05
rs17464492	128412048	0.21	0.04	0.08	0.92	0.09	6.14E-03	1.21E-02	0.72	1.18E-02	3.92E-04	4.80E-04	**6.42E-05**
rs10808555	128478693	6.35E-03	1.29E-02	0.81	0.05	0.05	3.87E-03	0.78	0.53	8.82E-03	**4.64E-05**	1.91E-04	**6.42E-05**
rs6983267	128482487	8.37E-03	**1.05E-05**	0.25	0.39	0.16	0.02	0.81	0.49	1.40E-03	**6.34E-08**	**1.28E-06**	8.33E-03
rs7837328	128492309	3.88E-03	8.68E-04	0.63	0.23	-	9.83E-03	0.84	0.62	1.41E-02	**4.40E-05**	3.02E-04	5.02E-03
rs7014346	128493974	6.19E-03	2.83E-03	0.69	0.18	0.23	0.02	0.98	0.49	1.02E-02	**8.48E-05**	6.32E-04	8.33E-03
rs12334695	128523110	0.14	4.34E-04	0.02	0.53	0.96	0.03	0.74	3.09E-02	0.47	**8.46E-05**	4.43E-04	8.33E-03
rs1447295	128554220	0.64	**2.44E-05**	0.52	0.46	0.24	0.17	0.27	0.12	0.73	1.26E-03	1.45E-02	0.61
rs4242382	128586755	0.83	**2.82E-06**	0.43	0.60	0.13	0.19	0.16	0.13	0.99	2.26E-04	0.06	0.61
rs4242384	128587736	0.87	**2.66E-06**	0.49	0.58	-	0.21	0.30	0.17	0.95	1.08E-03	0.11	0.57
rs7017300	128594450	0.73	**1.80E-05**	0.85	0.95	0.15	0.39	0.60	8.45E-02	0.58	2.03E-03	0.09	0.23
rs11988857	128601055	0.65	**3.78E-06**	0.97	0.87	-	0.16	0.37	5.68E-02	0.97	1.12E-03	0.29	0.19
rs7837688	128608542	0.88	**1.02E-06**	0.65	0.92	0.11	0.22	0.23	5.37E-02	0.74	**7.80E-05**	0.02	0.23
rs4404878	128867576	0.89	0.21	**3.02E-05**	0.06	0.50	0.05	0.17	0.10	0.97	3.20E-04	0.02	0.05
rs7841347	128887490	0.82	0.09	**3.25E-05**	0.06	0.38	0.54	0.27	0.25	0.86	7.96E-04	1.68E-02	0.05
rs16903097	129625538	0.59	0.92	0.39	0.05	-	1.98E-02	**9.16E-05**	0.14	0.33	1.08E-02	0.03	0.04
rs2163951	130672539	0.43	0.81	0.56	0.46	0.51	0.87	0.98	0.65	**9.72E-05**	0.03	0.35	0.61
rs6470863	132015998	0.55	0.32	0.87	**5.38E-05**	0.88	0.80	0.63	0.20	0.41	0.14	0.33	0.61

SNP markers with p ≤ .0001 (bold) for either the individual GWAS or the meta-analysis with SNP present in at least five studies, sorted based on base-pair position (bp) on chromosome 8. A dash indicates no data.

Results from ^1^CGEMS, ^2^Li et al [32], ^3^Song et al [35]; ^4^Permuth-Wey et al [36]; ^5^Wrensch et al [34]; ^6^CFR [33]

As shown in Table 2, rs6993464 is also the site of a weaker association peak in the CGEMS pancreatic cancer GWAS (p = 6.39E-05). However, the SNP rs6993464 retains a p-value of 7.94E-05 when the pancreatic cancer study is omitted from the analysis, suggesting that the association at rs6993464 is not solely driven by the pancreatic cancer study. Along with pancreatic cancer, the CGEMS lung and breast cancer datasets contribute the most to the significance of the meta-analysis p-values in this region, with study-specific p-values at these seven SNPs ranging from 0.008 to 5.45E-04. Unlike the CGEMS lung cancer GWAS, the never-smokers lung cancer GWAS did not contribute to the association signal at rs6993464 or nearby SNPs. This could be due to sample size, as the never-smokers dataset included fewer individuals (754 vs. 1629), or to gene-environment interaction, as the CGEMS lung cancer study included smokers, while the non-CGEMS lung cancer study did not.

An additional region consisting of 5 markers from 128,478,000 bp - 128,524,000 (around gene *POU5F1B*) had Fisher's p-values < 1E-04, including rs6983267, which had the smallest p-value in the meta-analysis (p = 6.34E-08) (Figure 1C). SNP rs10808555, and another SNP just outside the region (rs17464492; 128,412,048 bp), had p-values < 0.10 in 6 out of the 9 individual GWAS (binomial p = 6.4E-05). This result replicates previous reports of association between rs6983267 and prostate [20] and colon cancer [14-16,20], but provides conflicting evidence with respect to reports of association with ovarian cancer [20], as the UK ovarian study provided nominal support for association with rs6983267 (p = 0.022) while the US ovarian GWAS did not (p = 0.81).

Finally, two additional SNPs were found to be associated with cancer in the meta-analysis. An association at rs7837688 (128,608,542 bp; p = 7.80E-05) was primarily due to association with prostate cancer (individual study p = 1.02E-06). An association at rs6470494 (128,157,086 bp; p = 3.73E-05) showed some evidence of being a general cancer risk locus, as its significance was contributed by multiple data sets (colon, breast, and UK ovarian). However, this association was not as well supported by associations at neighboring SNPs.

This meta-analysis used equal weights for each cancer type; however, the studies included ranged in size from 350 subjects to 7174 subjects. As a sensitivity analysis, we also conducted a meta-analysis weighting each study by its sample size (Additional file 3). This analysis did not change the conclusions for rs6983267 (sample-weighted Fisher's p = 9.01E-05) or rs6993464 (p = 3.75E-07). However, the sample size-weighted analysis did result in a third region, 128,853 - 128,888 kb, showing p-values < 1E-04. The significance of this region was primarily due to the largest study, the pancreatic cancer data set. This region overlaps the gene *PVT1*, which has previously been implicated in breast and ovarian cancer [47]; however, in our analysis this region had nominal evidence of association with ovarian cancer (p = 0.017 at rs10956390 in the US ovarian data set) and no evidence of association with breast cancer.

## Discussion

Previous studies of the 8q24 region have identified associations between multiple types of cancer and markers around 128 Mb; in particular, rs6983267 has been associated with colon [20], prostate [14-16,20], and ovarian cancer [20]. This meta-analysis reproduces the association between rs6983267 and cancer, with four additional SNPs in the region 128,158,000 - 128,524,000 bp having Fisher's meta-analysis p-values < 1E-04. SNP marker rs6983267 has been shown to exhibit long-range physical interaction with the proto-oncogene c-MYC (about 335 kb downstream) in colorectal, prostate, and breast cancer [1,24], providing a potential mechanism for the source of this association.

**Figure 2:**
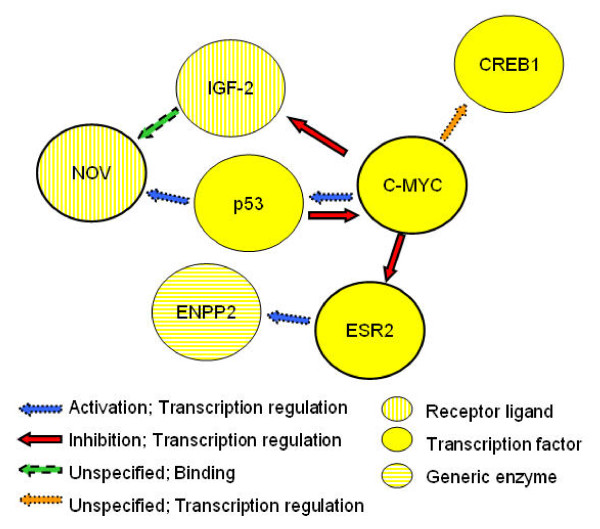
**Regulatory relationship between *NOV*, *ENPP2*, and *c-MYC*, reconstructed using MetaCore**.

In addition to the association at rs6983267, we identified a second SNP, rs6993464, associated with cancer risk. This SNP is not in LD with rs6983267 or other loci that have previously been reported to be associated with various cancers (Additional file 1). It is possible that the association at rs6993464 is due to several distinct variants, each of which influences a different type of cancer, rather than a single locus that influences cancer development for multiple cancers. This result would also be of interest, as it would imply the existence of previously unreported cancer-specific variants in 8q24. As shown in Figure 1B, rs6993464 and six other SNPs with meta-analysis p-values < 1E-04 lie between the genes *NOV *(nephroblastoma overexpressed gene) and *ENPP2 *(ectonucleotide pyrophosphatase/phosphodiesterase 2). *NOV *encodes the regulatory protein CCN3, which plays an important role in cancer development [26]. *ENPP2 *encodes a phospholipase which stimulates tumor cell motility and catalyzes the production of lysophosphatidic acid, which stimulates cell proliferation [48]. *ENPP2 *has also been reported to have angiogenic properties and its expression is up-regulated in several kinds of carcinomas [49]. Furthermore, MetaCore (GeneGo Inc., St. Joseph, MI) regulatory network analysis [50] indicated that both *NOV *and *ENPP2 *are indirectly regulated by the 8q24 proto-oncogene *c-MYC *(Figure 2), via *p53 *(for *NOV*) [51,52] and *ESR2 *(for *ENPP2*) [53,54], indicating their potential involvement in a pathway for cancer susceptibility. Therefore, it is functionally plausible that this region contains a locus for general cancer susceptibility via *cis *regulation of *NOV *and/or *ENPP2*.

It is also possible that this region harbors an enhancer locus for a more distant oncogene. Several of the significant SNPs in this region are found in the SCAN database [55] as expression quantitative trait loci (eQTLs) for genes throughout the genome which are associated with various types of cancer. In particular, rs6993464 has been shown to be an eQTL for *POLR2F*, a gene on chromosome 22 which is up-regulated in colorectal cancer [56], while rs7000665 regulates expression of *BRAF*, a gene which plays an important role in tumorigenesis in thyroid cancer [57] and melanoma [58], as well as in survival in colon cancer [59]. Finally, rs7846200 has been reported to be an eQTL for multiple genes associated with cancer, including *PTBP1*, which affects the invasive capacity of cancer cell lines in a cell type-dependent manner [60], and *HNRNPK*, which interacts with the oncogene p53 and contributes to pancreatic cancer [61] and has been associated with prostate cancer [62].

An additional locus, rs11987056, which is in perfect LD with rs6993464 (r^2 ^= 1), is highly conserved and only 170 bp downstream from the putative transcription factor binding sites V$S8_01 and V$NKX25_02 using the Transfac Matrix Database (v.7.0) [63]. This SNP is associated with expression of the proto-oncogene SRC, which is involved in the regulation of cell growth [64]. The SNP rs11987056 was not included in this analysis as it was genotyped in only two of the individual studies examined. Nonetheless, it is a strong functional candidate for the source of the association between rs6993464 and cancer development. Hence, there are several plausible mechanisms by which a locus in chromosome 8: 120,576,000 - 120,626,000 could affect general cancer susceptibility, explaining our meta-analysis findings.

Because Fisher's and Stouffer's tests are union-intersection tests [65], the meta-analysis peak at rs6993464 is not sufficient to conclude that the SNP is associated with multiple cancer types, only that it is associated with at least one type of cancer. However, the fact that rs6993464 retains a p-value of 7.94E-05 when pancreatic cancer, the study with the strongest association, is omitted from the analysis suggests that this locus is associated with more than one type of cancer in our study. Loci that affect general cancer susceptibility may act in a context-dependent manner; based on genetic background at other loci, tissue type, and other environmental factors, a particular variant could predispose for some types of cancers, while protecting against others. P-value based meta-analyses, in combination with two-sided tests of association such as those used the studies included here, are powered to detect opposite-directional associations. Among the three data sets which contributed the most significance at rs6993464, the T allele was associated with cancer risk in breast and pancreatic cancer, but with a protective effect in the CGEMS lung cancer data set. In the future, functional studies to establish the causal variant(s) that result in the association peak at rs6993464 may determine whether the opposite-directional association is due to context-dependent effects or to LD with distinct, tumor-specific modifiers.

A limitation of the current study is that the CGEMS lung and prostate cancer studies both drew samples from the PLCO Trial, and therefore the sets of controls overlapped. The pancreatic controls also included some individuals from PLCO (n ≤ 423), which may have overlapped with the lung and prostate cancer studies, and some controls from the Nurses' Health Study (n ≤ 166), which may overlapped with the breast cancer controls. Overlapping sets of controls could lead to inflation of -log_10 _p-values when the results are combined. However, when the prostate and pancreatic studies were omitted from the meta-analysis, thus removing any overlap in controls, rs6993464 retained a p-value of 6.89E-05. Similarly, marker rs6983267 had a p-value of 6.57E-08 when overlapping controls were removed by omitting the lung and pancreatic cancer data sets. This suggests that the overlapping sets of controls do not affect the overall conclusion of possible cancer risk loci around rs6983267 and rs6993464.

In the future, it would be beneficial to extend this analysis to include bladder cancer, another cancer type which has been associated with 8q24 [11]. It would also be advantageous to examine rs6993464 in the context of cancer-specific GWAS, conditioning on other loci which have been reported to be associated with these cancer types. This could clarify the role of rs6993464 among other loci that influence cancer susceptibility. This study demonstrates the power of meta-analysis for secondary phenotypes in identifying loci that may affect general cancer susceptibility. In the future, it would be valuable to conduct meta-analyses of subsets of solid-tumor cancer types with additional common features, such as breast, pancreatic, and colorectal cancer, which share the feature of frequent somatic amplification of 8q24 [20,25].

## Conclusions

In summary, this meta-analysis of nine existing GWAS for solid-cancers indicates a possible cancer risk locus on 8q24 (120,576,000-120,626,000 bp) between *NOV *and *ENPP2*, both of which are involved in carcinogenesis and have a regulatory relationship with the proto-oncogene *c-MYC*. We were also able to replicate previous findings for rs6983267, which has been implicated in risk for multiple cancers and known to have long-range physical interaction with the proto-oncogene *c-MYC*. Future research in this area is warranted to determine the mechanism by which this region may influence general cancer risk, as well as its genetic and environmental interactions with other known risk factors.

## Competing interests

The authors declare that they have no competing interests.

## Authors' contributions

AB performed the meta-analyses and drafted the manuscript. YWA performed the MetaCore and Transfac Matrix Database analyses. BLF conceived of the experiment and assisted in performing the meta-analyses and drafting the manuscript. HS, Y-YT, JAA, PY, RJ, PP, FS, DC, DJD, MJ, JH, SG, PN, GC, and TAS provided data and assisted in drafting the manuscript. All authors read and approved the final manuscript.

## Pre-publication history

The pre-publication history for this paper can be accessed here:

http://www.biomedcentral.com/1471-2350/12/156/prepub

## Supplementary Material

Additional file 1 **Location of SNPs previously reported to be associated with cancer**. The novel association, rs6993464, is indicated by the purple star. P-value plotted for previously reported associations is minimum from previous reports. Dark blue color indicates r^2 ^< 0.2. Shape of points indicates type of cancer with which the locus has been associated; there is no symbol for ovarian cancer because rs6983267, rs10808556, and rs10505477 have been associated with multiple cancer types (downward-pointing triangles). Lung cancer has been associated with rs6983267 (multiple cancer associations, downward-pointing triangle) and deletion D8S272 (137.8 Mb, not shown). Click here for file

Additional file 2 **Power of weighted and unweighted meta-analyses**. Three studies of size 350, 350, and 5000 were simulated according to a logistic model with log(OR) = Beta*x, where x is the genotype (-1, 0, 1). Power at alpha = 0.05 (over 1000 simulations) is shown for Fisher's meta-analyses with weights proportional to study size and with equal weights. Click here for file

Additional file 3 **Effect of sample size weighting on meta-analysis results**. SNP markers with p ≤ .0001 (bold) for the Fisher or Stouffer meta-analysis, weighted by cancer type or sample size. SNPs are sorted based on base-pair position (bp) on chromosome. Click here for file
