# Supplementary figures and images for: Meta-analysis of 8q24 for seven cancers reveals a locus between NOV and ENPP2 associated with cancer development
Source: BMC Med Genet. 2011 Dec 5;12:156. doi: 10.1186/1471-2350-12-156 (PMC3267702; doi:10.1186/1471-2350-12-156)

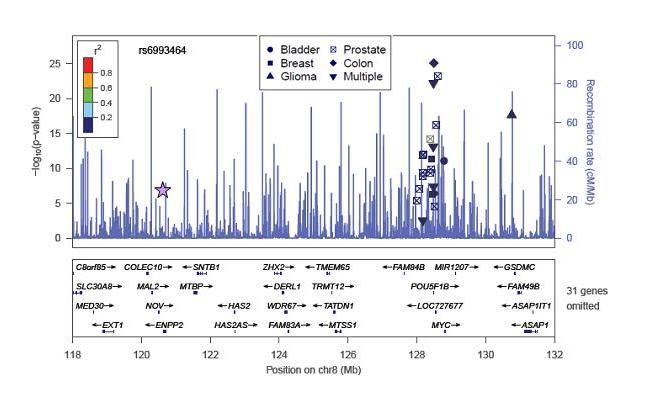

Supplement: Additional file 1 — Location of SNPs previously reported to be associated with cancer. The novel association, rs6993464, is indicated by the purple star. P-value plotted for previously reported associations is minimum from previous reports. Dark blue color indicates r2 < 0.2. Shape of points indicates type of cancer with which the locus has been associated; there is no symbol for ovarian cancer because rs6983267, rs10808556, and rs10505477 have been associated with multiple cancer types (downward-pointing triangles). Lung cancer has been associated with rs6983267 (multiple cancer associations, downward-pointing triangle) and deletion D8S272 (137.8 Mb, not shown). [file 1471-2350-12-156-S1.JPEG]
